# Prevention of Intraabdominal Adhesions: An Experimental Study Using Mitomycin-C and 4% Icodextrin

**DOI:** 10.4274/balkanmedj.2015.1359

**Published:** 2017-01-05

**Authors:** Murat Urkan, İsmail Hakkı Özerhan, Aytekin Ünlü, Mehmet Fatih Can, Erkan Öztürk, Armağan Günal, Gökhan Yağcı

**Affiliations:** 1 Department of Surgery, Gülhane Training and Research Hospital, Ankara, Turkey; 2 Department of Surgical Pathology, Gülhane Training and Research Hospital, Ankara, Turkey

**Keywords:** Cecal abrasion, intraabdominal adhesions, mitomycin-C, 4% icodextrin

## Abstract

**Background:**

Intraabdominal adhesions remain a significant cause of morbidity and mortality. Moreover, intraabdominal adhesions can develop in more than 50% of abdominal operations.

**Aims:**

We compared the anti-adhesive effects of two different agents on postoperative adhesion formation in a cecal abrasion model.

**Study Design:**

Experimental animal study.

**Methods:**

Forty Wistar albino type female rats were anesthetized and underwent laparotomy. Study groups comprised Sham, Control, Mitomycin-C, 4% Icodextrin, and Mitomycin-C +4% Icodextrin groups. Macroscopic and histopathological evaluations of adhesions were performed.

**Results:**

The frequencies of moderate and severe adhesions were significantly higher in the control group than the other groups. The mitomycin-C and Mitomycin-C +4% Icodextrin groups were associated with significantly lower adhesion scores compared to the control group and 4% Icodextrin group scores (p=0.002 and p=0.008, respectively). The adhesion scores of the Mitomycin-C group were also significantly lower than those of the 4% Icodextrin group (p=0.008).

**Conclusion:**

Despite its potential for bone marrow toxicity, Mitomycin-C seems to effectively prevent adhesions. Further studies that prove an acceptable safety profile relating to this promising anti-adhesive agent are required before moving into clinical trials.

Intraabdominal adhesions (IAs) after surgery are a common surgical problem that results in high mortality and morbidity rates (1,2). IAs are a major cause of complicated re-laparotomies, postoperative pain, and intestinal obstructions and have also been suggested as a possible cause of infertility in women (3,4,5). Literature data show that abdominal adhesions may develop after more than 50% of abdominal operations (6). Several surgical techniques, pharmacological agents and barriers have been proposed to reduce the incidence of IAs. Despite advances in the prevention and understanding of the underlying mechanisms of IAs, satisfactory improvements in clinical anti-adhesion protocols have not yet been established.

The adhesion formation is initiated by local injury on peritoneal plane during laparotomy. This local injury results in the production of an inflammatory exudate that comprises neutrophils, leukotriene-B4, interleukin-1 (IL-1) and IL-6, tumor necrosis factor, prostaglandin-E2, etc. in the peritoneal cavity. The exudate may act as an environment that enables fibroblasts to create fibrin bands connecting two adjacent visceral structures. Normally, these adhesions are attenuated by fibrinolytic mechanisms within three days. In the case of an augmented inflammatory response or prolonged tissue ischemia, the pathway may progress to fibrin deposition, organization and collagen formation, which in turn leads to IAs (7).

Mitomycin-C is a DNA alkylating antitumor antibiotic, which inhibits in vitro fibroblast proliferation with anti-fibrinolytic activity (8). Mitomycin-C has been used for strabismus surgery for the prevention of postoperative adhesion and recurrence of pterygium (9). Moreover, the intraperitoneal administration of Mitomycin-C has proven to be effective and safe for the prevention of primary or recurrent IAs in rats (10).

Icodextrin is a water-soluble branched glucose polymer linked by alpha 1-4 and 1-6 glucosidal bonds. Intraperitoneal administration of 4% Icodextrin acts as a resident colloidal osmotic agent. The colloidal osmotic action of this polymer retains a reservoir of fluid within the peritoneal cavity for 3-4 days, and creates a temporary physical separation between the peritoneal surfaces. This effect has been suggested to minimize surface apposition during the critical cycle of fibrin formation and reduces adherence formation (11).

The individual anti-adhesive properties of Mitomycin-C and 4% Icodextrin have been assessed by several studies. To the best of our knowledge, however, the combined anti-adhesive effects of these two agents have not been investigated. We hypothesized that Mitomycin-C and 4% Icodextrin would individually reduce IAs in a cecal abrasion model. We also hypothesized that the combined use of these two agents would also reduce IAs when compared to the control group, respectively.

## MATERIALS AND METHODS

The experimental study was approved by the Animal Care and Use Ethics Committee of Our Academy. All animals received humane care in accordance with the Guide for the Care and Use of Laboratory Animals published by the National Institute of Health (09 December 2011/11-63). Wistar albino female rats, weighing 250 to 300 g, were used in the study. All rats were quarantined for 1 week prior to the onset of study. All animals were kept under standardized conditions: temperature between 22 °C and 24 °C; relative humidity of 50-60%; and 12 h of light followed by 12 h of darkness. The animals had free access to food and water. Food was withdrawn 12 h before surgery and early in the postoperative period. All surgical interventions were carried out under general anesthesia using intramuscular 40 mg/kg ketamine hydrochloride (Ketalar^®^, Parke-Davis/Eczacıbaşı; Turkey) and 6 mg/kg Xylazine hydrochloride (Rompun^®^, Bayer; Mefar, Turkey). Sterile surgical technique was used throughout the intervention.

**Figure 1:**
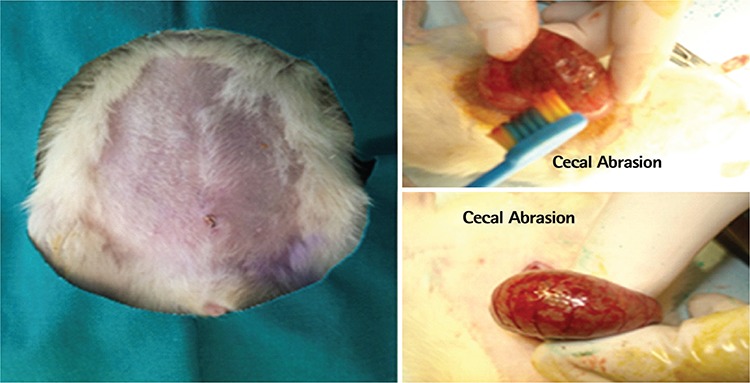
Application of experimental cecal abrasion model.

Forty female rats were randomly and evenly assigned into five study groups. We performed a laparotomy with a midline incision (3 cm). Afterwards, the cecum was exteriorized, approximately 1 cm^2^ of its anterior and medial serosae layer was denuded by brushing ten times with a sterile medium bristle tooth brush and returned to the abdominal cavity. Deserosalization was evidenced by punctate bleeding without hemostasis (Figure 1). Group 1, group 2, group 3, and group 4 (control) were administered intraperitoneal Mitomycin C (1 mg/kg) (Kyowa, Hakko, Kogyo, Co., Ltd.), 5 mL 4% Icodextrin (ADEPT^®^ Baxter; Deerfield, USA), Mitomycin-C + 4% Icodextrin combination, and 5 mL 0.9% NaCl, respectively. Group 5 (Sham) underwent only a laparotomy. No additional antibiotic was used during the experimental study. All animals were observed to assess the surgical complications.

**Figure 2:**
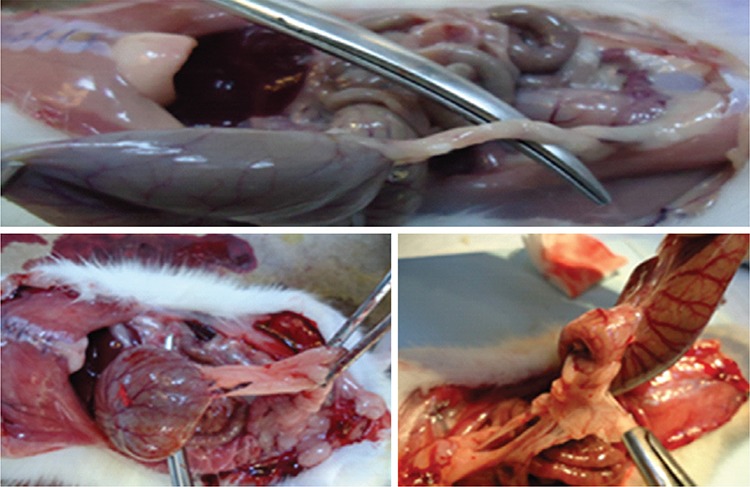
Typical adhesion of peritoneum after 21 days in the combined and control groups.

The rats were anesthetized and sacrificed 21 days after the initial surgery. The abdominal cavity was opened using a U-shaped incision for maximum exposure. The adhesion formation was evaluated according to the Nair adhesion scoring system by a surgeon blinded to the study groups (Table 1, Figure 2) (12). Tissue specimens were obtained from en-bloc excision of all adhesions around the cecum.

### Histopathological assessment

The specimens were fixed in 10% formalin and embedded in paraffin. Then, they were serially sectioned and stained with H&E. A blinded experienced pathologist examined the slides under light microscopy. The scoring system of Zühlke et al. (13) was used for the histopathological assessment of adhesions (Table 2).

The statistical analyses were performed using the SPSS statistical software package (version 15.0, IBM; USA). Differences between the study groups were analyzed using the chi-square test. Statistical significance was set at 0.05.

## RESULTS

There were two rats with local signs of wound infection during the experimental study. None of the rats died as a result of anesthesia or in the follow-up period.

Overall, the comparison of all study groups revealed statistically significant differences (p=0, 01) (Table 3). Macroscopically, the frequencies of moderate and severe adhesions were significantly higher in the control group than in the Mitomycin-C and Mitomycin-C +4% Icodextrin groups (p=0.002, p=0.008). The difference between the control group and the 4% Icodextrin group was not statistically significant (p=0.31). The mitomycin-C +4% Icodextrin group, however, was associated with significantly lower adhesion scores when compared to the control group (p=0.008). The Mitomycin-C +4% Icodextrin group was also associated with significantly lower adhesion scores than the 4% Icodextrin group (p<0.001). The Mitomycin-C group showed significantly lower adhesion scores when compared to the 4% Icodextrin group (p=0.008) (Table 4). Although the frequency of adhesion scores was lower in the Mitomycin-C +4% Icodextrin group than in the Mitomycin-C group, the difference was not statistically significant (p=0.31).

The differences between the histopathological adhesion scores of all groups were statistically significant (p=0.01) (Table 5). As expected, the Mitomycin-C and Mitomycin-C +4% Icodextrin group adhesion scores were significantly lower than those of the control group (p=0.006, p=0.004). However, the adhesion scores of the 4% Icodextrin group were not significantly different to those of the control group (p=0.88). The adhesion score of the Mitomycin-C group was also significantly lower than the 4% Icodextrin group (p=0.006) (Table 6).

## DISCUSSION

Despite the advances in surgical technique and use of widely available anti-adhesive agents, recent studies have showed that the postoperative intra-articular (IA) related burden on healthcare system are still considerably high. Postoperative adhesions have been reported to account for more than 40% of all cases with intestinal obstruction affecting the small bowel primarily in 60 to 70 percent of these cases (2,14,15).

Various strategies have been proposed to prevent adhesion formations, which include pharmacological agents, and a meticulous surgical approach with new equipment and instrumentation. The currently available knowledge indicates that three main methods have the potential to reduce postoperative IAs (10): (I) a reduction of peritoneal trauma by using minimally invasive approaches; (II) the use of pharmacological agents to intercept fibrin formation (heparin, tissue plasminogen activator (t-PA) or Mitomycin-C); and (III) reducing contact between intraabdominal organs and de-peritonized structures after dissection by using film or liquid barriers (Seprafilm^®^, Adept^®^). However, none of these approaches have been adopted as a standard therapy, and research for the ultimate solution still continues (16,17).

Tander et al. (18) and Cubukçu et al. (10) investigated Mitomycin-C for the prevention of IAs using experimental models; both studies showed that Mitomycin-C effectively prevented the formation of adhesions. Additionally, the application of 0.02% Mitomycin-C in topical eye solutions has been widely used in eye surgery for strabismus to reduce postoperative adhesions and in dacryocystorhinostomy to prevent obstruction of the common lacrimal canaliculus (19,20). Mitomycin-C has also been investigated for the prevention of pterygia recurrence after excision (21). It has become an agent of choice with favorable results in reducing postoperative scar formation (19). In accordance with the above-mentioned studies, the results of our macroscopic assessment showed a tendency towards adhesion reduction in the Mitomycin-C group. Additionally, histopathological examination of Mitomycin-C group specimens showed significantly lower adhesion scores.

Some studies show that 4% Icodextrin reduces the incidence of primary IAs and their reformation by separating the damaged area during the sensitive phase of recovery (15,22,23). Verco et al. (22) investigated 4% Icodextrin in a pre-clinical study and reported that Icodextrin (20 mL/kg) was successful in preventing IAs. Catena et al. (23) also studied the safety and efficacy of 4% Icodextrin for the prevention of IAs. The authors concluded that the application of 4% Icodextrin to extensively de-serosalized surfaces was more convenient when compared to other anti-adhesive barriers (22,23,24). In contrast to the above findings, Ditzel et al. (25) showed that 4% Icodextrin was not effective in significantly reducing the formation of adhesions. Additionally, Bellon et al. (26) investigated 4% Icodextrin and also failed to show a significant reduction in IAs. In the present study, our results also suggest that the individual use of 4% Icodextrin does not significantly contribute to adhesion prevention.

To our knowledge, experimental studies that investigate the combined use of anti-adhesive agents/barriers are scant. Kaptanoglu et al. (27) investigated the combined use of heparin and hyaluronic acid. They reported that the application of hyaluronic acid and heparin combination, showed no additive impact on reducing postoperative adhesion formation. Irkorucu et al. (28) compared the possible individual and combined effects of phosphatidylcholine (PC), hyaluronic acid and t-PA for the prevention of postoperative adhesion formation. They showed that use of the t-PA and PC combination was significantly superior to the individual use of PC, t-PA, and hyaluronic acid. Likewise, the results of the present study demonstrated that the combined use of Mitomycin-C +4% icodextrin resulted in significantly lower macroscopic and microscopic adhesion scores when compared to other groups.

In conclusion, despite its potential for bone marrow toxicity, Mitomycin-C seems to effectively prevent IAs. Moreover, our data suggest that the combined use of Mitomycin-C and 4% Icodextrin may be a better alternative to the single use of Mitomycin-C in preventing adhesions. Further studies that prove an acceptable safety profile relating to this promising antiadhesive agent are required before moving onto clinical trials.

## Figures and Tables

**Table 1:**
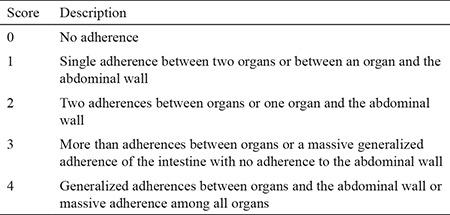
The grading criteria of Nair et al. (12) for adhesions in rats

**Table 2:**
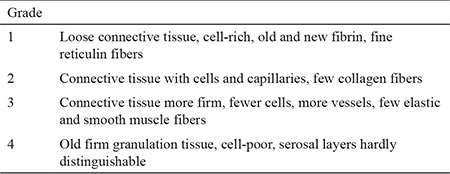
Histopathological classification according to Zühlke et al. (13)

**Table 3:**
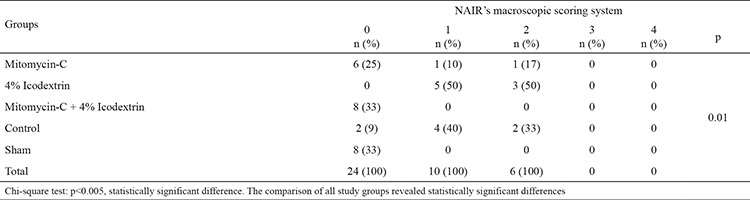
Groups with NAIR score system (statistically)

**Table 4:**
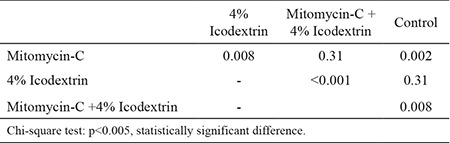
Macroscopic comparisons between groups statistically (p values)

**Table 5:**
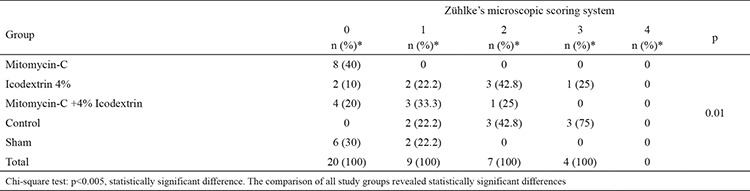
Compare the groups with Zühlke score system (statistically)

**Table 6:**
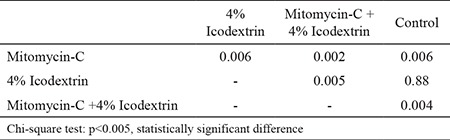
Microscopic comparisons between groups statistically (p values)
